# Patient and hospitalization differences in incarcerated versus nonincarcerated men: Insights from a 10-year cohort study

**DOI:** 10.1002/jhm.70297

**Published:** 2026-02-26

**Authors:** Farah Acher Kaiksow, Amir Forati, Kristin Merss, Karen Reece, Marguerite Burns, Eduard Eric Vasilevskis

**Affiliations:** 1 Department of Medicine, Division of Hospital Medicine, University of Wisconsin School of Medicine and Public Health, Madison, Wisconsin, USA; 2 Center for Aging Research and Education, University of Wisconsin School of Nursing, Madison, Wisconsin, USA; 3 Nehemiah Center for Urban Leadership Development, Madison, Wisconsin, USA; 4 Department of Population Health Sciences, University of Wisconsin School of Medicine and Public Health, Madison, Wisconsin, USA

## Abstract

**Background:**

The incarcerated population in the United States is underserved and aging rapidly; there is a dearth of information regarding their health, including hospital care. Epidemiological information is crucial to guide policymakers’ planning. This analysis provides comparison data on the admissions of incarcerated and nonincarcerated patients.

**Objectives:**

Identify differences in inpatient care between incarcerated and non-incarcerated patients by comparing 10 years of data at an academic medical center that serves as the largest single provider of hospital care for the state’s Department of Corrections.

**Methods:**

Ten-year cohort study comparing all admissions of incarcerated males (*n* = 4525) to a matched cohort of nonincarcerated (*n* = 13,575) at an academic medical center serving as the largest provider of hospital care for the state’s Department of Corrections. We included patients ≥18 years old and admitted to the hospital while in custody of the Department of Corrections between January 1, 2010, and December 31, 2019. Primary outcomes were patient characteristics, including demographics and comorbidities, and hospitalization characteristics, including length of stay and diagnosis.

**Results:**

Compared with the nonincarcerated cohort, the incarcerated patients were more likely to be Black (33.4% vs. 6.6%, *p* < 0.05), Hispanic/Latine (6.1% vs. 2.1%, *p* < 0.001), admitted to a general medicine service (33.4% vs. 22.6%, *p* < 0.01), and less likely to be admitted to neurological/psychiatric services (5.2% vs. 9.1%, *p* < 0.01). The incarcerated cohort had a higher mean Charlson Comorbidity Index (3.83 vs. 3.65, *p* < 0.001), with statistically significant differences observed among patients aged 40–59.

**Conclusion:**

In this cohort study, there were differences in patient and hospitalization characteristics between incarcerated and nonincarcerated patients that may have clinical implications. Policymakers and researchers must work toward improving both the health and health care of this marginalized population.

## INTRODUCTION

The United States is one of the world leaders in incarceration, with almost two million people in US prisons and jails on an average day.^1^ There are significant racial, ethnic, and socioeconomic inequities in who is incarcerated, with minoritized groups and those with less socioeconomic influence—groups that also have poorer health^2,3^—experiencing far greater rates of incarceration.^1^ People who are incarcerated thus face greater medical complexity and higher rates of many medical conditions.^4^ Compared with nonincarcerated, individuals in US prisons and jails have higher odds of chronic conditions, including hypertension, as well as many types of cancer, including cervical and lung cancers.^5,6^ Incarceration itself can worsen health; incarcerated individuals receive less treatment for chronic conditions such as hyper-tension and have higher cancer-related mortality.^7,8^

The mechanism by which incarceration impacts health is complex and multi-factorial, though the overall effect is that incarceration leads to accelerated aging and shortened life expectancy.^9^ In one study, people incarcerated in jails had the same prevalence of geriatric conditions at an average age of 59 as community-based adults aged 75 or over.^10^ This accelerated aging has significant implications for policy-making and resource allocation as carceral health care systems need to plan for geriatric care for individuals as young as 50 years.^11^

Compounding this challenge is the fact that the average age of the incarcerated population, like the broader US population, is increasing. Between 1999 and 2016, the number of incarcerated adults in the US aged 55 years or over increased by 280%; in 2016, this was nearly 160,000 people in state prisons alone.^12,13^

Information on the health care of incarcerated patients, such as conditions, costs, and outcomes, is limited.^14,15^ What is certain, however, is that as the incarcerated population ages, it will require more health care, including more frequent hospitalizations. Two previous studies described reasons for and characteristics of hospitalizations of incarcerated individuals, one reporting on 197 admissions between 2014 and 2017^16^ and the second detailing 204 admissions in 2018.^17^ Conclusions from these studies are limited by their sample sizes, differences in data collection methods used for the two samples, and the lack of comparison to nonincarcerated patients.^16^

The current study updates and adds to the existing knowledge base by comparing 10 years of data on hospitalizations of incarcerated patients to nonincarcerated patients at an academic medical center that serves as the largest single provider of hospital care for the state’s Department of Corrections (DOC). By comparing admissions of incarcerated to nonincarcerated patients, we aimed to identify potential differences in both patient and hospitalization characteristics.

## METHODS

### Study design

We conducted a cohort study to compare the characteristics of hospitalizations for males who were incarcerated in a state DOC facility to an age- and admission year-matched cohort of non-incarcerated males admitted to the same hospital.

### Study sample

We used the electronic medical record (EMR) of a large, academic, Level I trauma center. We identified patients who were male, at least 18 years old, and admitted to the hospital for any reason while in the custody of the DOC between January 1, 2010, and December 31, 2019. We chose this timeframe to obtain a continuous 10-year period that avoided the coronavirus pandemic. We chose to exclude females from our analysis, given the significant differences in their health profiles compared with males who are incarcerated, such as higher rates of mental health diagnoses^18^ and a higher burden of chronic illness.^19^ We included patients who were admitted to our hospital’s dedicated DOC, or “locked,” unit, as well as patients admitted to other units. As the largest single provider of hospital care for our state’s DOC, our hospital has a locked unit with seven rooms that can hold up to 10 incarcerated patients at one time. Security services are provided by DOC officers, while health care services are provided by the general hospital staff. There are no nurses, physicians, or other members of the health care team who provide care solely in the locked unit; any member of the hospital staff can be called upon to see incarcerated patients, either in the locked unit or elsewhere. Decisions on where to place incarcerated patients in the hospital are based on medical conditions and level of care needed, and are unrelated to the security level (minimum, medium, maximum) of the patient’s carceral facility. In general, only patients requiring a general level of care, rather than a critical or intermediate level of care, are placed in the locked unit.

Because there is currently no consistent or agreed-upon method of identifying incarcerated patients in the EMR, our dataset included six variables that could support a patient’s incarceration status (please see Supporting Information S1: Appendix 1): Private hospital encounter flag, DOC “locked” hospital unit, patient address (e.g. address of the DOC), guarantor address or name (e.g., DOC address), billing account address or name, and Insurance payor or benefit plan name. We considered patients incarcerated if they at least two of six EMR indicators of DOC custody status.

These criteria resulted in a sample of 4525 admissions over the 10-year period. To create a comparison cohort, we matched non-incarcerated patients on age, gender, and admission year in a 3:1 ratio (*n* = 13,575). Because our goal was to evaluate for potential associations between incarceration status and our chosen outcome measures, we matched based on age to minimize its contribution to the outcomes. Our analysis included both inpatient and observation admissions.

### Variables

The primary exposure variable was incarceration status. Outcome variables were demographic characteristics, comorbidity burden (as measured by the Charlson Comorbidity Index, or CCI^20^), primary hospital diagnoses as administratively coded on discharge using the 10th revision of the International Classification of Diseases (ICD-10) codes,^21^ and initial admitting service using admission orders (please see Supporting Information S1: Appendix 2 for a complete list of all admitting services). We extracted patient and hospitalization characteristics from the EMR for both the exposed (incarcerated) and nonexposed (nonincarcerated) groups. We conducted all data analysis at the admission level; each admission of a patient who was admitted more than once during the 10-year period was counted as a separate hospitalization and analyzed independently.

#### Patient characteristics

Sociodemographic characteristics included age, race (American Indian or Alaska Native, Asian, Black or African American, and White), and ethnicity (Hispanic/Latine and Not Hispanic/Latine). We created a category for patients with more than one race (>1 Race).* Health characteristics included individual comorbidities as administratively coded on discharge using ICD-10 codes and the CCI, as previously described. For ease of analysis, ICD-10 codes were collapsed into clinically meaningful categories; for example, all substance use disorders were collapsed into one grouping, all depression codes were collapsed into another grouping, and the remaining mental health diagnoses were collapsed into a third grouping (please see Supporting Information S2: Appendix 3 for full details).

#### Hospitalization characteristics

Hospital variables included admitting service, primary hospital diagnosis, length of stay in days, and 30-day readmission rate to the same hospital. To simplify analysis and interpretation, we collapsed admitting service into fewer categories, including Medicine, Surgery, and Neurology/Psychiatry (please see Supporting Information S1: Appendix 4 for the final categorization of admitting services). We collapsed Neurology, Epilepsy, Psychiatry, and Stroke into a single category given their clinical overlap in terms of symptoms, diagnoses, and treatment approaches. To protect patient privacy, we excluded services that included 10 or fewer admissions over the 10 years. We noted whether incarcerated patients were hospitalized in our hospital’s forensic (locked) unit or in a standard patient room.

### Statistical analysis

We designed the analytic plan to assess differences between incarcerated and nonincarcerated patient populations using appropriate inferential methods. To accomplish this, we first compared patient demographics and hospitalization characteristics, primary hospital diagnoses, and admitting service information, all stratified by incarceration status. For continuous variables, including age and length of stay, we used independent two-sample *t*-tests to evaluate mean differences between groups. Before applying the *t*-test, we tested the assumption of homogeneity of variances with Levene’s test and assessed normality with the Shapiro–Wilk test. For categorical variables, such as the distribution of primary hospital diagnoses and comorbidities, we used *z*-tests for proportion differences. The *z*-test was used to assess whether the observed differences in proportions between incarcerated and nonincarcerated groups were statistically significant, providing insight into disparities across various patient characteristics.

Following the demographic comparisons, we further stratified the cohorts by age groups (<40, 40–49, 50–59, 60–69, 70–79, and ≥80 years) for ease of analysis and because the CCI does not change linearly. We computed the mean CCI for each age group over the 10-year study period and subjected it to comparative analysis between incarcerated and nonincarcerated cohorts. We implemented adjustments for multiple comparisons to mitigate the risk of Type I error. This study was approved by the University of Wisconsin-Madison Institutional Review Board.

## RESULTS

### Patient characteristics

**FIGURE 1:**
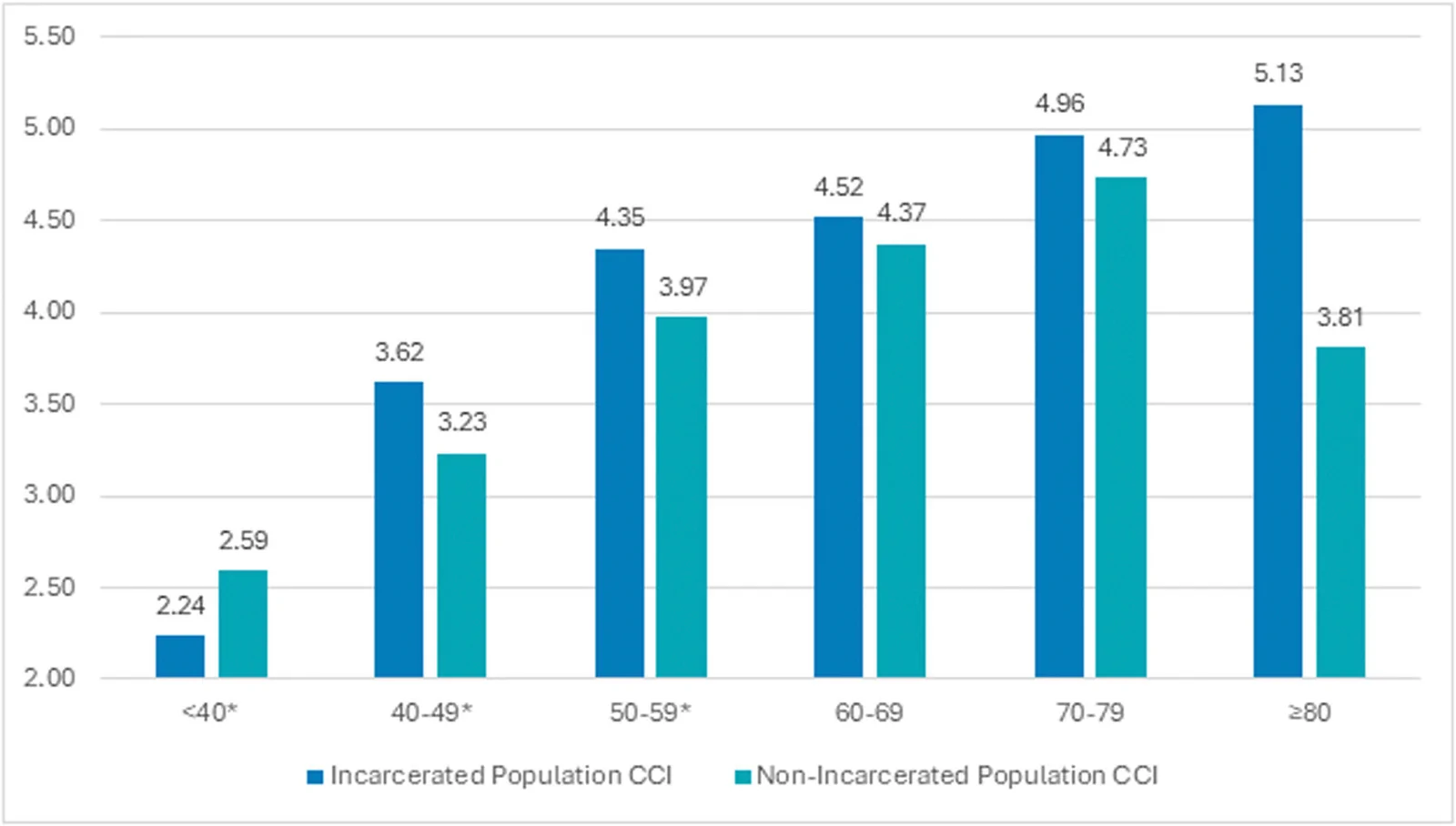
Average Charlson Comorbidity Index by age group. This figure illustrates the average Charlson Comorbidity Index (CCI) scores across different age groups. The CCI is a method of predicting mortality by classifying or weighting comorbidities. Age groups are categorized as follows: <40, 40–49, 50–59, 60–69, 70–79, and 280. Statistically significant differences (at the *p* < 0.05 level) were found between groups of patients aged <40, 40–49, and 50–59 (marked with an asterisk [*]).

Our final age-matched sample included 4525 incarcerated and 13,575 nonincarcerated inpatients (Table 1). The mean age of the overall study cohort was 47.7 years and, due to intentional matching, was equivalent in both groups. Incarcerated inpatients were more likely to be from historically marginalized groups, including American Indian/Alaska Native (2.9% incarcerated vs. 0.7% nonincarcerated, *p* < 0.001), Black or African American (33.4% vs. 6.6%, *p* < 0.05), and Hispanic/Latine (6.1% vs. 2.1%, *p* < 0.001). The mean length of stay was 0.5 days longer for incarcerated inpatients. There was no difference in 30-day readmission rates. Over one-third of incarcerated patient hospitalizations occurred in standard (not locked) hospital rooms; even after removing admissions to intensive care-level services, one-third of the remaining hospitalizations for incarcerated patients were outside the locked unit. The mean CCI was consistently higher for incarcerated individuals across all age groups beginning at age 40, with statistically significant differences observed in the 40–49 and 50–59 age groups (Figure 1).

### Hospitalization characteristics

There were differences in admitting service patterns (Table 2). Incarcerated patients were more frequently accepted to medicine services (52.9% for incarcerated vs. 41.3% for nonincarcerated, *p* < 0.01), while nonincarcerated patients were more frequently accepted to surgical (40.6% vs. 46.5%, *p* < 0.01) and neurological/psychiatric (1.3% vs. 3.1%, *p* < 0.01) services. Within medicine services, incarcerated patients were more likely to be admitted to general medicine/hospitalist services (33.4% vs. 22.6%, *p* < 0.01), rather than specialty services such as cardiology. There were minor differences in primary hospital diagnoses (Table 3), with the most notable being the much lower proportion of incarcerated patients admitted for “mental, behavioral, and neurodevelopmental disorders” compared with nonincarcerated (2.1% vs. 7.6%, *p* < 0.01).

## DISCUSSION

In the largest cohort of incarcerated patients yet studied, we examined 10 years of data from an academic medical center that serves as the largest provider of hospital care for the state’s DOC and found key differences in patient- and hospitalization-level characteristics when compared with an age-matched nonincarcerated group. Incarcerated patients aged ≥40 years had higher comorbidity burden, with the largest differences occurring among middle age, specifically for groups aged 40–49 and 50–59. Length of stay and readmissions rates for the two groups, however, were similar. Incarcerated patients were less likely to be admitted to specialty or surgical services compared with nonincarcerated. We also found that over one-third of hospitalizations for incarcerated patients occurred outside of our hospital’s designated forensic unit. Taken together, our findings provide guidance for health systems and Departments of Corrections to address the needs of the rapidly aging incarcerated population.

We found that incarcerated patients were more likely to come from racial and ethnically marginalized populations; this finding is not unexpected given the well-established inequities in racial and ethnic incarceration rates in the United States.^22^ Our finding that incarcerated patients experience a higher comorbidity burden compared with age-matched controls is also in line with previous studies reporting more medical complexity and accelerated aging among incarcerated individuals.^4,10^ In our study population, for example, incarcerated patients aged 50–59 had almost the same CCI as the nonincarcerated cohort aged 60–69. The increased illness severity among incarcerated patients supports recent calls for increased attention to and funding for this rapidly aging and underserved population, in terms of clinical care, research, and broader policy adjustments.^23–28^

Given the higher CCI among incarcerated patients, our finding that the groups had the same 30-day readmission rates is unexpected. Although initially developed to predict 1-year mortality, the CCI has also been shown to predict short-term readmissions for multiple conditions, including heart failure and orthopedic surgery.^29–31^ Unfortunately, we do not have information on which conditions resulted in readmissions in our cohort and so cannot draw conclusions on what the appropriate readmission rates might be for each group, but the higher CCI for the incarcerated patients suggests their rates should have been higher that what was found. Future efforts to determine whether there are differences in admissions for preference-sensitive^32^ conditions between incarcerated and non-incarcerated patients would provide significant insight into where variability in readmission decision-making for these two groups occurs. Such work may also help identify sources of bias, such as at the carceral provider, health system provider, or health system level.^33^

More than a third of all admissions of incarcerated patients occurred outside of our locked forensic unit. To our knowledge, this distinction has not been quantified before, and it has implications for patient care. Patients outside the locked unit may benefit from being placed in a standard room; their providers face lower barriers to entry and may thus receive higher quality, more frequent care.^34^ On the other hand, incarcerated patients in our locked unit experience more privacy and potentially more therapeutic rest as carceral officers are not required to be in the rooms with the patients at all times. There are also implications for health care systems; as the population ages, these systems need to be prepared to manage an increasing number of admissions of incarcerated patients, many of which are likely to occur outside of traditional locked units. This evolution will create both physical and personnel challenges, requiring potential alterations to hospital environments and additional training for clinical staff.

There were differences in patterns of admissions to inpatient services that were not explained by differences in hospital diagnoses. A larger proportion of incarcerated patients were admitted to general medicine services, with fewer admitted to specialty cardiology services such as electrophysiology and advanced heart failure. This particular difference in care patterns may negatively influence the health of the incarcerated population given previous studies showing that specialized heart failure care is associated with improved outcomes.^35–37^ Similarly, an explanation for the large difference in admission to medical versus surgical services cannot be found in differences in primary hospital diagnoses, and there is no evidence to suggest that incarcerated individuals require fewer surgeries than nonincarcerated.^38^ Given the tendency for undifferentiated patient cases to be admitted to general rather than specialized services, this difference in admission patterns may itself be a sign of inadequate pre-admission evaluation and triage. It may also be a marker of health care provider bias toward incarcerated patients, which can further exacerbate health inequities.^33^

One final consideration is the potential under-coding among incarcerated patients’ hospitalization records. Although not yet studied in the incarcerated population, analogous research regarding EMR data—whether missing or inaccurate—suggests that the burdens of these errors fall disproportionately on people of lower socioeconomic status.^39,40^ In our study, the low or relatively low rates of certain comorbidities coded for incarcerated inpatients raises the question of under-coding. Mental health conditions provide an illustrative example. Although the lack of data makes it difficult to know the true number of incarcerated patients who suffer from SUD, prevalence estimates range from 18% to 30% for alcohol misuse and 10%–48% for drug misuse.^41^ Similarly, rates of depression and other mental health diagnoses among incarcerated patients are estimated to be two to four times that of the nonincarcerated population.^42^ These numbers contrast with our data, in which SUD was coded fewer than half as many cases and depression was coded 15% less often among incarcerated compared with nonincarcerated inpatients. Although differences between our data and previous reports may be partially explained by the data sources, as much of the previously published work in this field comes from patient self-report, objective data from both the US Department of Justice and the National Institute on Drug Abuse suggest that our findings do indeed indicate an under-coding of both SUD and other mental health diagnoses.^43,44^ Particularly for SUD, the implications of this under-coding may be severe; hospitalization is a key opportunity for initiation of medications for opioid use disorder.^45^ Failure to recognize and/or document the condition, however, represents a significant barrier to treatment for this marginalized population.

### Limitations

Our study has limitations. First, there are inherent limitations to using EMR data, with multiple studies highlighting the inaccuracies found in EMRs.^46,47^ As discussed, incarcerated patients may face higher rates of EMR inaccuracies, including in the documentation of comorbidities. Our data suggest that EMR data likely underestimates incarcerated patient comorbidities, meaning that incarcerated patients are sicker than their charts would indicate. Additionally, the CCI is not an independent measurement but is associated with many other patient comorbidities listed in Table 1 and may also be related to a patient’s hospital diagnosis. CCI also fails to include many diagnoses that may be particularly relevant to incarcerated patients, including mental health conditions. We elected not to analyze mortality as using only our EMR data would have underestimated the mortality rate for incarcerated patients. Similarly, our data did not include information on procedures, meaning we were unable to determine if the higher proportion of patients triaged to medicine services over surgical services represented fewer surgeries for these patients, or simply a co-management model. Finally, we do not know if any of the controls in our sample had a history of incarceration; even after release, strong evidence supports the impact of incarceration on health persists.^9^

Besides the limitations of an EHR study, our study has other limitations. We faced challenges in accurately identifying patients who were hospitalized while incarcerated. Although we believe our refined criteria are accurate for our institution, there is currently no validated method to identify hospitalizations of incarcerated individuals, and, given differences in health care systems and EMRs, there is unlikely to be a process that can be universally applied across institutions. Second, we chose not to match based on documented race or ethnicity. The relationship between incarceration and race is complex; race may act as a confounder or as a moderator, and there may be an interactive effect of being both from a minoritized group and being incarcerated. If we chose to match on race, we would eliminate that interaction, which may have significant policy implications. Because male individuals make up over 90% of all people incarcerated in the United States, we elected to include only male patients in our study. Our data should not be used to make conclusions regarding females who are incarcerated, particularly given the known differences in health profiles between the two groups.^18,19^ Additionally, this was a single-site study, and our findings may not be generalizable to other health systems or other states.

## CONCLUSION

This cohort study provides the most comprehensive data yet on the hospitalizations of incarcerated men in the United States compared with an age-matched group of nonincarcerated patients. There is a growing population of older incarcerated adults in the United States, and our study shows that they are sicker than the overall population; our health care system must be prepared for the increasing demands of these patients. Potential next steps for policymakers include an increased focus on geriatric care and geriatric care providers within carceral systems, evaluation of geriatric and compassionate release programs, and alterations to some prison environments to improve accessibility for an aging population. Future research efforts should focus on improving identification of incarcerated patients, assessing for biases in comorbidity coding and documentation within EMRs, and differentiation between care provided in locked units compared with standard patient rooms. Caring for the incarcerated population is a public responsibility, and action to address this looming public health crisis should begin now.

## Supplementary Material

jhm70297-sup-0001-supplementary_materials_1

SUPPORTING INFORMATION

Additional supporting information can be found online in the Supporting Information section at the end of this article.

## Figures and Tables

**TABLE 1:** Characteristics of hospitalizations for age-matched male incarcerated and non-incarcerated patients, 2010–2019.

Characteristic	Incarcerated, no. (%) (total = 4525)	Nonincarcerated, no. (%) (total = 13,575)	*p*
Mean age (years)	47.7	47.7	n/a
Race			
American Indian/Alaska Native	123 (2.9%)	97 (0.7)	<0.001
Asian	27 (0.6)	130 (1.0)	<0.05
Black	1421 (33.4)	899 (6.6)	<0.05
Native Hawaiian/Other Pacific Islander	2 (0.1)	16 (0.1)	0.17
White	2,879 (67.7)	12,165 (89.6)	<0.05
>1 Race	38 (0.9)	146 (1.1)	0.17
Unknown/Blank	35 (0.8)	122 (0.9)	0.043
Ethnicity			
Hispanic/Latine	277 (6.1)	288 (2.1)	<0.001
Not Hispanic/Latine	4194 (92.7)	13,196 (97.2)	<0.001
Unknown/Blank	54 (1.2)	91 (0.7)	<0.001
Comorbidities^a^			
Any comorbidity	2361 (52.2)	7897 (58.2)	<0.001
Diabetes	767 (17.0)	2086 (15.4)	<0.05
Dementia	17 (0.4)	72 (0.5)	0.20
Hypertension	1535 (33.9)	4417 (32.5)	0.09
Intellectual disability	10 (0.2)	270 (2.0)	<0.001
Depression	858 (19.0)	3073 (22.6)	<0.001
Other mental health diagnosis	180 (4.0)	550 (4.1)	0.83
Obesity	384 (8.5)	1724 (12.7)	<0.001
Substance use	494 (10.9)	3212 (23.7)	<0.001
Mean CCI	3.83	3.65	<0.001
Length of stay (days)			
Mean	4.4	3.9	<0.001
30-day readmission			
Yes	662 (14.63%)	2029 (14.95%)	0.604
In locked unit			
Yes	2891 (63.9)		
No	1634 (36.11)		

*Note*: Numbers do not sum to the total number of discharges as each discharge may have more than one comorbidity associated with it. Please see Supporting Information S1: Appendix S1 for a list of ICD-10 codes associated with each comorbidity.

a Comorbidities as coded on hospital discharge.

**TABLE 2:** Distribution of admitting services for incarcerated and nonincarcerated patients, 2010–2019.^a^

Admitting service	Incarcerated, no. (%) (total = 4462)	Nonincarcerated, no. (%) (total = 12,486)	*p*
Medicine			
General medicine/Hospitalist	1488 (33.4%)	2819 (22.6%)	<0.01
Cardiology (general)	398 (8.9)	987 (7.9)	0.04
Hematology/Oncology	255 (5.7)	634 (5.1)	0.11
Critical care	160 (3.6)	396 (3.2)	0.20
Cardiac electrophysiology	27 (0.6)	139 (1.1)	<0.01
Cardiac catheterization	21 (0.5)	86 (0.7)	0.14
Advanced heart failure	11 (0.3)	94 (0.8)	<0.01
All medicine	2360 (52.9)	5155 (41.3)	<0.01
Surgery			
General surgery	397 (8.9)	1083 (8.7)	0.67
Neurosurgery	258 (5.8)	751 (6.0)	0.60
Orthopedic surgery	212 (4.8)	978 (7.8)	<0.01
Urology	207 (4.6)	580 (4.7)	1.0
Otolaryngology	170 (3.8)	321 (2.6)	<0.01
Transplant	104 (2.3)	471 (3.8)	<0.01
Plastic surgery	91 (2.0)	148 (1.2)	<0.01
Trauma surgery	87 (2.0)	554 (4.4)	<0.01
Vascular Surgery	77 (1.7)	289 (2.3)	<0.05
Thoracic surgery	49 (1.1)	101 (0.8)	0.09
Cardiac surgery	38 (0.9)	160 (1.3)	<0.05
Ophthalmology	37 (0.8)	53 (0.4)	<0.01
Colorectal surgery	32 (0.7)	71 (0.6)	0.33
Surgical critical care	17 (0.4)	59 (0.5)	0.51
Minimally invasive surgery	11 (0.3)	12 (0.1)	<0.05
Burn	14 (0.3)	141 (1.1)	<0.01
Interventional radiology	11 (0.3)	37 (0.3)	0.71
All surgery	1812 (40.6)	5809 (46.5)	<0.01
*Neurology/psychiatry*			
Neurology	130 (2.9)	221 (0.8)	<0.01
Stroke	52 (1.2)	143 (1.2)	0.98
Psychiatry	28 (0.6)	757 (6.1)	<0.01
Epilepsy	24 (0.5)	16 (0.1)	<0.01
All neurology/psychiatry	234 (5.2)	1137 (9.1)	<0.01
*Blank*	56 (1.3)	385 (3.1)	<0.01

a Totals vary from Tables 2 and 3 as specialties with <10 admissions were excluded (Clinical Research Study, Endocrine, Neurocritical Care, Oncologic Surgery, Outpatient Service, Palliative Care, Pediatric Cardiology, Pediatric Neurosurgery, Pediatric Otolaryngology, Pediatric Plastic Surgery, Pediatric Trauma, Pulmonary, Rehabilitation Medicine).

**TABLE 3:** Primary hospital diagnosis as classified by ICD-10 category, 2010–2019.

ICD-10 class	Incarcerated, no. (%) (total = 4525)	Nonincarcerated, no. (%) (total = 13,575)	*p*
Diseases of the digestive system	687 (15.2%)	1479 (10.1%)	<0.01
Injury, poisoning and certain other consequences of external causes	677 (15.0)	2228 (16.4)	0.14
Diseases of the circulatory system	566 (12.5)	2104 (15.5)	<0.05
Neoplasms	428 (9.5)	1021 (7.5)	<0.01
Diseases of the musculoskeletal system and connective tissue	270 (6.0)	865 (6.4)	0.51
Certain infectious and parasitic diseases	219 (4.8)	601 (4.4)	0.49
Diseases of the respiratory system	206 (4.6)	685 (5.1)	0.46
Diseases of the genitourinary system	187 (4.1)	584 (4.3)	0.79
Diseases of the nervous system	149 (3.3)	446 (3.3)	1.0
Endocrine, nutritional, and metabolic diseases	135 (3.0)	489 (3.6)	0.24
Diseases of the skin and subcutaneous tissue	127 (2.8)	292 (2.2)	0.14
Diseases of the blood and blood-forming	104 (2.3)	143 (1.1)	<0.01
Mental, behavioral, and neurodevelopmental disorders	94 (2.1)	1026 (7.6)	<0.01
Diseases of the ear and mastoid process	37 (0.8)	95 (0.7)	0.51
Diseases of the eye and adnexa	31 (0.7)	54 (0.4)	.012
Other^a^	528 (11.7)	1321 (9.7)	<0.01
Not available^b^	80 (1.8)	142 (1.1)	0.07

a Other includes: (1) Congenital malformations, deformations, and chromosomal abnormalities; (2) Symptoms, signs, and abnormal clinical and laboratory findings, not elsewhere classified; and (3) Factors influencing health status and contact with health services.

b Not available includes ICD-10 codes IMO0001 and IMO0002.

## Footnotes

* The authors acknowledge that race and ethnicity are social constructs and have no biological basis or impact on health. We use them here as a marker of the potential impact of societal factors on the patient.
