# Supplementary material for: Patient and hospitalization differences in incarcerated versus nonincarcerated men: Insights from a 10‐year cohort study
Source: J Hosp Med. 2026 Feb 26;21(10):1115–23. doi: 10.1002/jhm.70297 (PMC13051773; doi:10.1002/jhm.70297)
Supplement: Supplementary file 1 [file JHM-21-1115-s001.docx]

Appendix 1. Electronic medical record fields used to identify patients who were hospitalized while incarcerated

| **Field** | **Value** |
| --- | --- |
| Private Hospital Encounter Flag | “Security Patient” |
| Hospital Unit | Value for hospital forensic/locked unit |
| Patient Address | Value included known carceral facility address |
| Guarantor Address or Name |  |
| Billing Account Address or Name |  |
| Insurance Payor or Benefit Plan Name | Value included carceral facility name |

Appendix 2. All admitting services

Advanced heart failure

Blank

Burn

Cardiac catheterization

Cardiac electrophysiology

Cardiac surgery

Cardiology (general)

Clinical research study

Colorectal surgery

Critical care

Endocrine

Epilepsy

General medicine/

General surgery

Hematology/Oncology

Hospitalist

Interventional radiology

Minimally invasive surgery

Neurocritical care

Neurology

Neurosurgery

Oncologic surgery

Ophthalmology

Orthopedic surgery

Otolaryngology

Outpatient service

Palliative care

Pediatric cardiology

Pediatric neurosurgery

Pediatric otolaryngology

Pediatric plastic surgery

Pediatric trauma

Plastic surgery

Psychiatry

Pulmonary

Rehabilitation medicine

Stroke

Surgical critical care

Thoracic surgery

Transplant

Trauma surgery

Urology

Vascular Surgery

Advanced heart failure

Blank

Appendix 3. ICD-10 Codes used to create comorbidity categories in Table 1

| **Category** | **ICD-10 Codes** |
| --- | --- |
| Diabetes mellitus | E10-E14 |
| Dementia | F01*, F02*, F03*, G30* |
| Hypertension | I10-I15 |
| Intellectual disability | F70X-F79X, F80X-F89X |
| Depression | F20X-F29X, F30-F39X, F40-F48X |
| Other mental health diagnosis | F06X, F07X, F09X, F50X-59X, F60X-69X, F90X-F98X, F99X |
| Obesity | E66-E66.9 |
| Substance use disorder | F10X-F19X |

Appendix 4. Admitting service categories (collapsed)^a^

| **Admitting service** | **Services included** | |
| --- | --- | --- |
| **Medicine** | | |
| General medicine/  Hospitalist | Teaching Services  General medicine 1A  General medicine 1B  General medicine 2A  General medicine 2B  General medicine 3A  General medicine 3B | Non-teaching services  Hospital medicine  Hospitalist med svc 1  Hospitalist med svc 2 Hospitalist med svc 3  Hospitalist med svc 4  Hospitalist med svc 5  Hospitalist med svc 6 |
| Cardiology (general) | Teaching Services  Cardiovascular medicine red  Cardiovascular medicine blue | Non-teaching services  Cardiology  Cardiology_old |
| Hematology/Oncology | Bone marrow transplant  Hematology  Hematology/Oncology  Oncology | |
| Advanced heart failure | Advanced heart failure  Congestive heart failure | |
| **Surgery** | | |
| General surgery | Emergency general surgery  General surgery  Surgery | |
| Neurosurgery | Neurosurgery  Neurosurgery_old | |
| Orthopedic surgery | Orthopedic surgery  Orthopedic surgery_old | |
| Urology | Urology  Urology_old | |
| Transplant | Cardiac transplant  Liver transplant  Medical transplant  Pancreas transplant  Renal transplant  Thoracic transplant  Transplant cardiology | |
| Ophthalmology | Ophthalmology  Ophthalmology_old | |
| Burn | Burn  Burn_old | |
| **Other** | | |
| Neurology | Neurology  Neurology_old | |

^a^If a category is not listed, it was not combined with any other categories in the final analysis.
