# Supplementary material for: Patient and hospitalization differences in incarcerated versus nonincarcerated men: Insights from a 10-year cohort study
Source: J Hosp Med. Author manuscript; Available in PMC 2026 Apr 6. (PMC13051773; doi:10.1002/jhm.70297)
Supplement: jhm70297-sup-0001-supplementary_materials_1 [file NIHMS2155748-supplement-jhm70297-sup-0001-supplementary_materials_1.docx]

Appendix 1. Electronic medical record fields used to identify patients who were hospitalized while incarcerated

| **Field** | **Value** |
| --- | --- |
| Private Hospital Encounter Flag | “Security Patient” |
| Hospital Unit | Value for hospital forensic/locked unit |
| Patient Address | Value included known carceral facility address |
| Guarantor Address or Name |  |
| Billing Account Address or Name |  |
| Insurance Payor or Benefit Plan Name | Value included carceral facility name |

Appendix 2. All admitting services

| Advanced heart failure |
| --- |
| Blank |
| Burn |
| Cardiac catheterization |
| Cardiac electrophysiology |
| Cardiac surgery |
| Cardiology (general) |
| Clinical research study |
| Colorectal surgery |
| Critical care |
| Endocrine |
| Epilepsy |
| General medicine/ |
| General surgery |
| Hematology/Oncology |
| Hospitalist |
| Interventional radiology |
| Minimally invasive surgery |
| Neurocritical care |
| Neurology |
| Neurosurgery |
| Oncologic surgery |
| Ophthalmology |
| Orthopedic surgery |
| Otolaryngology |
| Outpatient service |
| Palliative care |
| Pediatric cardiology |
| Pediatric neurosurgery |
| Pediatric otolaryngology |
| Pediatric plastic surgery |
| Pediatric trauma |
| Plastic surgery |
| Psychiatry |
| Pulmonary |
| Rehabilitation medicine |
| Stroke |
| Surgical critical care |
| Thoracic surgery |
| Transplant |
| Trauma surgery |
| Urology |
| Vascular Surgery |
| Advanced heart failure |
| Blank |

Appendix 3. ICD-10 Codes used to create comorbidity categories in Table 1

| **Category** | **ICD-10 Codes** |
| --- | --- |
| Diabetes mellitus | E10-E14 |
| Dementia | F01*, F02*, F03*, G30* |
| Hypertension | I10-I15 |
| Intellectual disability | F70X-F79X, F80X-F89X |
| Depression | F20X-F29X, F30-F39X, F40-F48X |
| Other mental health diagnosis | F06X, F07X, F09X, F50X-59X, F60X-69X, F90X-F98X, F99X |
| Obesity | E66-E66.9 |
| Substance use disorder | F10X-F19X |

Appendix 4. Admitting service categories (collapsed)^a^

| **Admitting service** | **Services included** | |
| --- | --- | --- |
| **Medicine** | | |
| General medicine/  Hospitalist | Teaching Services  General medicine 1A  General medicine 1B  General medicine 2A  General medicine 2B  General medicine 3A  General medicine 3B | Non-teaching services  Hospital medicine  Hospitalist med svc 1  Hospitalist med svc 2 Hospitalist med svc 3  Hospitalist med svc 4  Hospitalist med svc 5  Hospitalist med svc 6 |
| Cardiology (general) | Teaching Services  Cardiovascular medicine red  Cardiovascular medicine blue | Non-teaching services  Cardiology  Cardiology_old |
| Hematology/Oncology | Bone marrow transplant  Hematology  Hematology/Oncology  Oncology | |
| Advanced heart failure | Advanced heart failure  Congestive heart failure | |
| **Surgery** | | |
| General surgery | Emergency general surgery  General surgery  Surgery | |
| Neurosurgery | Neurosurgery  Neurosurgery_old | |
| Orthopedic surgery | Orthopedic surgery  Orthopedic surgery_old | |
| Urology | Urology  Urology_old | |
| Transplant | Cardiac transplant  Liver transplant  Medical transplant  Pancreas transplant  Renal transplant  Thoracic transplant  Transplant cardiology | |
| Ophthalmology | Ophthalmology  Ophthalmology_old | |
| Burn | Burn  Burn_old | |
| **Other** | | |
| Neurology | Neurology  Neurology_old | |

^a^If a category is not listed, it was not combined with any other categories in the final analysis.
